# On the structure of mirrored operators obtained from optimal entanglement witnesses

**DOI:** 10.1038/s41598-023-37771-0

**Published:** 2023-07-03

**Authors:** Anindita Bera, Joonwoo Bae, Beatrix C. Hiesmayr, Dariusz Chruściński

**Affiliations:** 1 Institute of Physics, Faculty of Physics, Astronomy and Informatics, Nicolaus Copernicus University, Grudzia̧dzka 5/7, 87–100 Toruń, Poland; 2 School of Electrical Engineering, Korea Advanced Institute of Science and Technology (KAIST), 291 Daehak-ro, Yuseong-gu, Daejeon, 34141 Republic of Korea; 3 Faculty of Physics, University of Vienna, Währingerstrasse 17, 1090 Vienna, Austria

**Keywords:** Mathematics and computing, Physics

## Abstract

Entanglement witnesses (EWs) are a versatile tool in the verification of entangled states. The framework of mirrored EW doubles the power of a given EW by introducing its twin—a mirrored EW—whereby two EWs related by mirroring can bound the set of separable states more efficiently. In this work, we investigate the relation between the EWs and its mirrored ones, and present a conjecture which claims that the mirrored operator obtained from an optimal EW is either a positive operator or a decomposable EW, which implies that positive-partial-transpose entangled states, also known as the bound entangled states, cannot be detected. This conjecture is reached by studying numerous known examples of optimal EWs. However, the mirrored EWs obtained from the non-optimal ones can be non-decomposable as well. We also show that mirrored operators obtained from the extremal decomposable witnesses are positive semi-definite. Interestingly, the witnesses that violate the well known conjecture of Structural Physical Approximation, do satisfy our conjecture. The intricate relation between these two conjectures is discussed and it reveals a novel structure of the separability problem.

## Introduction

Entanglement witnesses (EWs) are a both theoretical and experimental tool to detect entangled states^1–6^. When an entangled state $\rho$ realized in experiment is identified by quantum state tomography, there exists an EW that finds if it is entangled, i.e.,

1 $$\begin{aligned} \textrm{tr}[W\rho ]<0,~\textrm{whereas}~ 0\le \textrm{tr}[W\sigma _{\textrm{sep}}],~~\forall ~\sigma _{\textrm{sep}}\in \textrm{SEP} ~~~ \end{aligned}$$

where $\textrm{SEP}$ denotes the set of separable states. In fact, Eq. (1) can be used as a definition of entangled states: a bipartite state $\rho$ is entangled if and only if there exists an EW *W* such that $\textrm{tr}[W \rho ] <0$, and therefore *W* detects, i.e. *witnesses* the entanglement^1^.

Since EWs correspond to the Hermitian operators, they can be realized experimentally for the verification of entangled states. This also means that entanglement can be directly verified in experiment without the identification of a given state, i.e. by in general, less measurement setups. In general, an EW can be decomposed into local observables,

2 $$\begin{aligned} W = \sum _{i,j} c_{ij} A_i\otimes B_j, \end{aligned}$$

with some numbers of $c_{ij}$ and local observables $A_i$ and $B_j$. A collection of expectation values of local observables $\langle A_i \otimes B_j \rangle _{\rho } = \textrm{tr}[A_i\otimes B_j \rho ]$ so that one computes $\textrm{tr}[W \rho ] = \sum _{ij} c_{ij} \langle A_i\otimes B_j\rangle _{\rho }$ detects if a state $\rho$ is entangled.

Due to the well known Choi–Jamiołkowski isomorphism^7,8^, there is one to one correspondence between the block-positive operators in ${\mathcal {H}}_A \otimes {\mathcal {H}}_B$ and positive maps ${\mathcal {B}}({\mathcal {H}}_A) \rightarrow {\mathcal {B}}({\mathcal {H}}_A)$, where ${\mathcal {B}}({\mathcal {H}})$ denotes bounded linear operators acting on ${\mathcal {H}}$ (in this paper we consider only finite dimensional Hilbert spaces). Entanglement witnesses correspond to positive but not completely positive maps^9–11^.

Optimal EWs are of particular importance^12^. An EW *W* is called optimal if $W - \epsilon P$ for all non-negative operators $P\ge 0$ and for any $\epsilon >0$ is no longer an EW. That is, one cannot improve *W* by subtracting a positive operator.

Entanglement witnesses, being Hermitian operators, represent physical observables and hence in principle, can be implemented in the laboratory. However, positive maps which are not completely positive are not physically realizable. The idea of structural physical approximation (SPA) is to mix a positive map with an amount of the completely depolarizing map as small as possible in order to obtain a physically realizable completely positive map^13,14^. Equivalently, SPA to an entanglement witness *W* is defined by

3 $$\begin{aligned} X = p\; \mathbbm {1}_A \otimes \mathbbm {1}_B + W, \end{aligned}$$

with the smallest $p>0$ such that $X \ge 0$ (i.e. $p = - \lambda _{\textrm{min}}$, where $\lambda _{\textrm{min}}$ is a minimal eigenvalue of *W*). Note that a SPA operator *X* may also be interpreted as a not normalized quantum state.

The SPA conjecture in Ref.^15^ has asserted that SPA to an optimal EW leads to a separable state *X*, or, equivalently, SPA to optimal positive trace-preserving map leads to entanglement-breaking quantum channels (cf. also Refs.^16,17^ and Ref.^18^ for an review). It was firstly supported by many examples of optimal EWs^18–21^, however, later it was disproved^22–24^.

In this paper, we consider a similar concept based on the notion of a mirrored operator introduced in Ref.^25^. Given an EW *W*, we define a mirrored operator by

4 $$\begin{aligned} W_{\textrm{M}} = \mu \mathbbm {1}_A \otimes \mathbbm {1}_B - W, \end{aligned}$$

with the smallest $\mu >0$ such that $W_{\textrm{M}}$ is block-positive, i.e. $\langle \psi \otimes \phi |W_{\textrm{M}}|\psi \otimes \phi \rangle \ge 0$. Moreover, if the maximal eigenvalue of *W* satisfies $\lambda _{\textrm{max}} > \mu$, then $W_{\textrm{M}}$ is an EW and hence one has a pair $(W,W_{\textrm{M}})$ of mirrored EWs^25^, which can double up the capability of detecting entangled states. This framework is also referred to as “*entanglement witnesses 2.0*” since every witness comes with another one, in analogy to software programs that improve with each new version.

An important property of EWs is its (non)-decomposibility. An EW is called decomposable if $W=A + B^\Gamma$, with $A,B \ge 0$ and $\Gamma$ stands for the partial transposition. Note that decomposable witnesses cannot detect PPT entangled states, i.e. if $W = A + B^\Gamma$, then $\textrm{Tr}(W\sigma )\ge 0$ for all PPT states $\sigma$. This is easily proved by exploiting the fact that the trace is invariant under transposition, i.e. for a PPT state $\sigma$, $\textrm{Tr}(A\sigma )+\textrm{Tr}( B^\Gamma \sigma )=\textrm{Tr}(A\sigma )+\textrm{Tr}( B\sigma ^\Gamma ) \ge 0$, since $A,B\ge 0$ and $\sigma ,\sigma ^\Gamma \ge 0$.

In this paper, we investigate the structure of mirrored EWs. In particular, we address the following question: given an optimal EW, what are the properties of the corresponding mirrored one? As an answer, we propose a conjecture which says that given an optimal EW, its mirror operator $W_{\textrm{M}}$ is either a decomposable EW or just a positive operator. In other words, there does not exist a mirrored pair of non-decomposable EWs $(W,W_{\textrm{M}})$ such that at least one of them is optimal. The assumption about optimality is crucial. Actually, we show one can construct a mirrored pair of non-decomposable EWs but none of them is optimal. We believe that our analysis paves a new avenue of finding a fine structure of the set of entanglement witnesses and thus the structure of separable and PPT-entangled states in the Hilbert space.

The paper is organized as follows. In Section “Mirrored entanglement witnesses”, we discuss the concept of mirrored EWs and propose our conjecture. In Section “Optimal entanglement witnesses: basic properties”, we review the basic properties of optimal entanglement witnesses. Section “Mirroring optimal decomposable entanglement witnesses” provides the analysis of our conjecture for decomposable EWs. Moreover, we show that our conjecture holds true for a class of extremal decomposable EWs. In Section “Mirroring non-decomposable entanglement witnesses”, we provide several examples of optimal non-decomposable EWs supporting the above conjecture. Additionally, we construct an entanglement witness in ${\mathbb {C}}^4 \otimes {\mathbb {C}}^4$ which is non-decomposable and not optimal and find that the mirrored EW is non-decomposable as well. Interestingly, we include the analysis of optimal EWs which were used to disprove SPA conjecture^22,24^. Numerical analysis shows that both examples support our conjecture. We finally conclude in Section “Conclusions”.

## Mirrored entanglement witnesses

**Figure 1:**
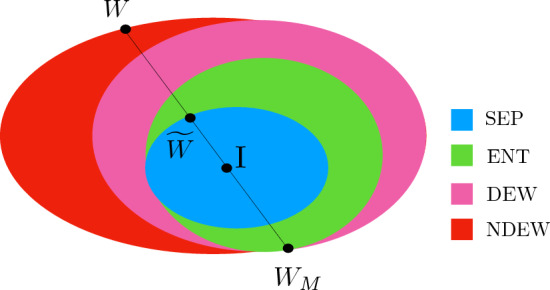
The sets of separable states (SEP), entangled states (ENT), decomposable EWs (DEWs), and non-decomposable EWs (NDEWs) are compared in the view of our conjecture and the SPA one. Given an NDEW *W*, SPA to *W* is denoted by ${\widetilde{W}}$ and $W_M$ is the corresponding mirrored witness. The SPA conjecture addresses that SPA to an optimal EWs are separable states. Counterexamples are, however, obtained in Refs.^22–24^. Our conjecture suggests that a mirrored operator $W_{\textrm{M}}$ obtained from an optimal EW is either a decomposable EW or a positive operator.

The concept of mirrored EW is closely related to SPA which we shortly summarize here (see Fig. 1). Given a bipartite operator $Q\ge 0$, let us define the followings:

5 $$\begin{aligned} a_-:= & {} \inf _{|\psi \otimes \phi \rangle } \, \langle \psi \otimes \phi | { Q }| \psi \otimes \phi \rangle , \nonumber \\ a_+:= & {} \sup _{|\psi \otimes \phi \rangle } \, \langle \psi \otimes \phi | { Q }| \psi \otimes \phi \rangle , \end{aligned}$$

where $|\psi \otimes \phi \rangle$ is normalized product vector in ${\mathcal {H}}_A \otimes {\mathcal {H}}_B$. Defining a pair of block-positive operators with the values obtained above

6 $$\begin{aligned} W_-:= & {} a_+ \mathbbm {1}_A \otimes \mathbbm {1}_B - Q, \nonumber \\ W_+:= & {} Q - a_- \mathbbm {1}_A \otimes \mathbbm {1}_B, \end{aligned}$$

or equivalently

7 $$\begin{aligned} W_+ + a_- \mathbbm {1}_A \otimes \mathbbm {1}_B = Q = a_+ \mathbbm {1}_A \otimes \mathbbm {1}_B - W_- \, \end{aligned}$$

one can say that a positive operator *Q* represents two complementary SPA $W_+$ and $W_-$, i.e a *positive * SPA $W_+$ and *negative* SPA to $W_-$.

Moreover, the paired EWs in (6) are related as follows:

8 $$\begin{aligned} W_+ + W_-= \mu \mathbbm {1}_A \otimes \mathbbm {1}_B, \end{aligned}$$

where $\mu = a_+ - a_-$. Hence, we can say that two EWs $W_+$ and $W_-$ are mirrored to each other, see also Eqs. (3) and (4). To clarify the above relations, the separability bounds of $W_+$ are

9 $$\begin{aligned} 0\le \textrm{tr}[W_+ \sigma _{\textrm{sep}}] \le \mu , ~~\forall \sigma _{\textrm{sep}}\in \textrm{SEP}, \end{aligned}$$

where the upper bound is equivalent to the condition $\textrm{tr}[ W_- \sigma _{\textrm{sep}} ]\ge 0$, the very definition of an entanglement witness. From Eq. (8), a reciprocal relation leads to the separability bounds for $W_-$,

10 $$\begin{aligned} 0\le \textrm{tr}[W_- \sigma _{\textrm{sep}}] \le \mu , ~~\forall \sigma _{\textrm{sep}}\in \textrm{SEP}, \end{aligned}$$

where the upper bound is equivalent to the condition $\textrm{tr}[ W_+ \sigma _{\textrm{sep}} ]\ge 0$.

Having introduced mirrored EWs, we are now ready to address the conjecture.

### Conjecture

*A mirrored operator obtained from an optimal EW is either a positive operator or a decomposable EW and hence cannot detect PPT-entangled states.*

Equivalently, the above conjecture asserts that there does not exist a pair of non-decomposable EWs $(W,W_{\textrm{M}})$ such that at least one of them is optimal. Similarly to the SPA conjecture, the conjecture mentioned above is concerned with the optimality of an EW and its mirrored one. The conjecture is motivated by the observation in Eq. (8) that shows a trade-off relation between EWs: if one is closer to the identity, the other one is further away from the identity. Our conjecture is that this observation is related to the optimality of EWs.

## Optimal entanglement witnesses: basic properties

Given an EW *W*, let us denote ${\mathcal {D}}_{W}$, by a subset of states detected by *W*^12^, i.e. a set of states $\rho$ such that $\textrm{tr}(W\rho ) < 0$. One calls $W_1$ is finer than $W_{2}$ if $D_{W_{2}} \subseteq D_{W_{1}}$. *W* is optimal if there is no finer EW than *W*. Optimality of *W* is equivalent to the following property^12^: if *W* is optimal, then $W-P$ is no longer an EW, where *P* is an arbitrary positive operator. It means that one cannot improve *W* (i.e. make it finer) by subtracting $P \ge 0$. Note that optimality does not protect to subtract a block-positive operator. Finally, *W* is extremal if and only if $W-B$ is no longer an EW, where *B* is an arbitrary block- positive operator such that $B \ne \lambda W$.

Clearly, any extremal EW is optimal. However, the converse needs not be true. An EW corresponding to so-called reduction map $R_n : M_n({\mathbb {C}}) \rightarrow M_n({\mathbb {C}})$ defined by

11 $$\begin{aligned} R_n(X) = \mathbbm {1}_n \textrm{tr}X - X, \end{aligned}$$

is optimal for all $n\ge 2$ but extremal only for $n=2$.

In general, given *W* it is very hard to check whether it is optimal. There exists, however, an operational sufficient condition for optimality^12^. Denote by $P_W$ a set of product vectors $|\psi \otimes \phi \rangle$ such that

12 $$\begin{aligned} \langle \psi \otimes \phi |W| \psi \otimes \phi \rangle = 0. \end{aligned}$$

One has the following^12^

### Proposition 1

*If* $\textrm{span}\, P_W = {\mathcal {H}}_A \otimes {\mathcal {H}}_B$, *then W is optimal*.

In this case, i.e. when $\textrm{span}~P_W = {\mathcal {H}}_A \otimes {\mathcal {H}}_B$, one says that *W* has the spanning property. It should be stressed, however, that there exists optimal EWs without spanning property (cf. recent discussion in Ref.^26^).

Consider a decomposable EW $W = A + B^\Gamma$ in ${\mathbb {C}}^n \otimes {\mathbb {C}}^m$. Recall that *W* is optimal if $W = B^\Gamma$ and *B* is supported on completely entangled subspace (CES)^12^. A linear subspace $\Sigma \subset {\mathbb {C}}^n \otimes {\mathbb {C}}^m$ defines a CES if it does not contain a product vector. It is well known that a maximal dimension of any CES in ${\mathbb {C}}^n \otimes {\mathbb {C}}^m$ is $(n-1)(m-1)$^27,28^ The simplest example of CES is a 1-dimensional subspace spanned by an arbitrary entangled vector $|\Psi \rangle \in {\mathbb {C}}^n \otimes {\mathbb {C}}^m$. The corresponding entanglement witness $|\Psi \rangle \langle \Psi |^\Gamma$ is extremal^4^. It is, therefore, clear that any decomposable EW is a convex combination of extremal witnesses.

For non-decomposable EWs, the situation is much more complicated^29^. Recall that a bipartite state is called a PPT state (Positive Partial Transpose) if $\rho ^\Gamma \ge 0$, i.e. both $\rho$ and $\rho ^\Gamma$ are legitimate quantum states. Now, *W* is a non-decomposable EW if and only if it detects a PPT-entangled state. Let ${\mathcal {D}}^{\textrm{PPT}}_W$ be a set of PPT states detected by *W*. Now, $W_1$ is non-decomposable–finer than $W_2$ if ${\mathcal {D}}^{\textrm{PPT}}_{W_2} \subseteq {\mathcal {D}}^{\textrm{PPT}}_{W_1}$. An EW *W* is non-decomposable–optimal if there is no non-decomposable–finer EW than *W*. Actually, if an EW *W* is non-decomposable–optimal, then $W-D$ for a PPT operator *D* is no longer an EW. It means that one cannot improve *W* (i.e. make it finer) by subtracting a PPT operator *P*. Interestingly, it has been proven^12^

### Proposition 2

*W is non-decomposable–optimal if and only if both W and* $W^\Gamma$ *are optimal*.

A similar concept but on the level of states is provided by so-called *edge state*^30^.

### Definition 1

A PPT-entangled state $\rho$ is called an edge state if $\sigma =\rho - \epsilon |\psi \otimes \phi \rangle \langle \psi \otimes \phi |$ is no longer a PPT operator for arbitrary product state $|\psi \otimes \phi \rangle$ and arbitrarily small $\epsilon$, i.e. either $\sigma$ or $\sigma ^\Gamma$ is not positive.

**Figure 2:**
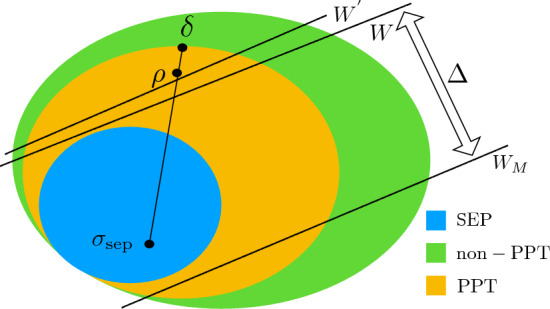
The set of quantum states is depicted. A PPT-entangled state $\rho$ can be expressed as a convex mixture of a separable state $\sigma _{\textrm{sep}}$ and an edge state $\delta$. EWs such as *W* or $W^{'}$, which are also non-decomposable, may detect an entangled state $\rho$. For mirrored EWs *W* and $W_{\textrm{M}}$, the separability window is denoted by $\Delta$.

It simply means that if $\rho$ is an edge state, then one cannot subtract any PPT state out of it without destroying a PPT property, as it is shown in Fig. 2. Authors of^30^ provided the following representation of non-decomposable EWs: let $\rho _{\textrm{edge}}$ be an edge state. To construct an EW detecting $\rho _{\textrm{edge}}$, consider two positive operators *P* and *Q* such that

$$\begin{aligned} \textrm{Ran}\,P \subseteq \textrm{Ker}\, \rho _{\textrm{edge}}\, \ \ \ \ \textrm{Ran}\,Q \subseteq \textrm{Ker}\, \rho _{\textrm{edge}}^\Gamma , \end{aligned}$$

where $\textrm{Ran}$ and $\textrm{Ker}$ denote the range and the kernel of the corresponding operator, respectively. Define

13 $$\begin{aligned} W = P + Q^\Gamma - \epsilon _-\, \mathbbm {1}_A \otimes \mathbbm {1}_B, \end{aligned}$$

with $\epsilon _- = \inf _{|\psi \otimes \phi \rangle } \, \langle \psi \otimes \phi | (P+Q^\Gamma )| \psi \otimes \phi \rangle$. By construction, *W* is block-positive and

14 $$\begin{aligned} \textrm{tr}(W \rho _{\textrm{edge}}) = \textrm{tr}[(P+Q^\Gamma )\rho _{\textrm{edge}}] - \epsilon _- = - \epsilon _- < 0, \end{aligned}$$

which shows that *W* is a non-decomposable EW detecting $\rho _{\textrm{edge}}$. Note that using Eq. (13), one can easily find the mirrored operator

15 $$\begin{aligned} W_{\textrm{M}} = \epsilon _+\, \mathbbm {1}_A \otimes \mathbbm {1}_B - (P + Q^\Gamma ), \end{aligned}$$

with $\epsilon _+ = \sup _{|\psi \otimes \phi \rangle } \, \langle \psi \otimes \phi | (P+Q^\Gamma )| \psi \otimes \phi \rangle$. However, it is not clear that whether the formula (15) provides a decomposable EW, non-decomposable EW or a positive operator. In what follows, we provide several examples of optimal non-decomposable EWs for which $W_{\textrm{M}}$ is never non-decomposable, i.e. it is either decomposable EW or a positive operator.

Finally, we observe that if $W_{\textrm{M}}$ is a mirrored operator to *W* i.e.

16 $$\begin{aligned} W_{\textrm{M}} = \mu \, \mathbbm {1}_A \otimes \mathbbm {1}_B - W, \end{aligned}$$

then

17 $$\begin{aligned} W^\Gamma _{\textrm{M}} = \mu \, \mathbbm {1}_A \otimes \mathbbm {1}_B - W^\Gamma , \end{aligned}$$

is a mirrored operator to $W^\Gamma$ with the same $\mu$.

## Mirroring optimal decomposable entanglement witnesses

Let us consider a decomposable EW in ${\mathbb {C}}^n \otimes {\mathbb {C}}^m$. We start our analysis with extremal decomposable EWs, i.e. $W=|\Psi \rangle \langle \Psi |^\Gamma$ for some entangled state $|\Psi \rangle \in {\mathbb {C}}^n \otimes {\mathbb {C}}^m$^4,5^.

### Proposition 3

*If* $W = |\Psi \rangle \langle \Psi |^\Gamma$, *then the mirrored operator* $W_{\textrm{M}}$ *is positive semi-definite*.

### Proof

Let

18 $$\begin{aligned} |\Psi \rangle = \sum _{k=0} s_k |e_k \otimes f_k \rangle , \end{aligned}$$

stand for the Schmidt decomposition of $|\Psi \rangle$ with $s_0 \ge s_1 \ge s_2 \ge {\cdot\cdot\cdot} \ge 0$. Note that the maximal eigenvalue of $|\Psi \rangle \langle \Psi |^\Gamma$ equals $s_0^2$ and it corresponds to the product vector $|e_0 \otimes f_0^*\rangle$. One has therefore

19 $$\begin{aligned} \mu = \sup _{|\psi \otimes \phi \rangle } \langle \psi \otimes \phi |\Psi \rangle \langle \Psi |^\Gamma | \psi \otimes \phi \rangle = s_0^2, \end{aligned}$$

and hence the mirrored operator

20 $$\begin{aligned} W_{\textrm{M}} = s_0^2 \, {\mathbb {I}}_n \otimes {\mathbb {I}}_m - |\Psi \rangle \langle \Psi |^\Gamma , \end{aligned}$$

is by construction positive definite. $\square$

In particular, if $|\Psi \rangle =|\Psi ^+_n\rangle$ is a maximally entangled state, i.e. $|\Psi ^+_n\rangle = \frac{1}{\sqrt{n}} \sum _{k=0}^{n-1} |k \otimes k\rangle$, then $s_0^2=1/n$ and hence

21 $$\begin{aligned} W_{\textrm{M}} = \frac{1}{n} \Big ( {\mathbb {I}}_n \otimes {\mathbb {I}}_n - {\mathbb {F}} \Big ) \ge 0, \end{aligned}$$

where ${\mathbb {F}}$ is a flip (swap) operator defined via

22 $$\begin{aligned} {\mathbb {F}} = n |\Psi ^+_n\rangle \langle \Psi ^+_n|^\Gamma = \sum _{i,j=0}^{n-1} |i\rangle \langle j| \otimes |j\rangle \langle i|. \end{aligned}$$

### Remark 1

Interestingly, if $W = |\Psi \rangle \langle \Psi |^\Gamma$, then the corresponding SPA is always a separable operator^15^. Hence, for extremal decomposable EWs both conjectures hold true.

Beyond the extremal EWs $W = |\Psi \rangle \langle \Psi |^\Gamma$, we do not have a proof of our conjecture. However, there are several examples supporting it.

### Example 1

Consider an EW corresponding to the reduction map (11)

23 $$\begin{aligned} W = {\mathbb {I}}_n \otimes {\mathbb {I}}_n - n P^+_n, \end{aligned}$$

with $P^+_n = |\Psi ^+_n\rangle \langle \Psi ^+_n|$ being the rank-1 projector onto canonical maximally entangled state. One easily finds $\mu =1$ and hence the mirrored operator

24 $$\begin{aligned} W_{\textrm{M}} = n P^+_n, \end{aligned}$$

is evidently positive definite. Moreover, SPA corresponding to Eq. (23) satisfies the SPA conjecture^15^.

### Example 2

In Ref.^24^, the authors have provided a family of decomposable witnesses in ${\mathbb {C}}^3 \otimes {\mathbb {C}}^3$ mentioned below which violate SPA conjecture:

25 $$\begin{aligned} W_\gamma =3B_{\gamma }^\Gamma , \end{aligned}$$

with

26 $$\begin{aligned} B_\gamma =\frac{1-\gamma }{2} P_{10}+\frac{1-\gamma }{2} P_{20}+\gamma P_{11}, \end{aligned}$$

where $P_{kl}=|\Omega _{kl} \rangle \langle \Omega _{kl}|$ denotes a set of rank-1 projectors with $|\Omega _{kl} \rangle =W_{kl} \otimes {\mathbb {I}}|\Omega _{00}\rangle$, $W_{kl}$ is a Weyl operator defined by $W_{kl} |i\rangle =w^{k(i-l)}|i-l\rangle$ with $w=e^{2\pi i/3}$. One can express $W_{\gamma }$ explicitly in the following matrix form

27 $$\begin{aligned} W_{\gamma }=\left( \begin{array}{ccccccccc} 1-\gamma &{}. &{}. &{}. &{}. &{}. &{}. &{} \gamma w &{}. \\ . &{} \gamma &{}. &{} -\frac{1-\gamma }{2}&{}. &{}. &{}. &{}. &{}. \\ . &{}. &{}. &{}. &{} \gamma w^* &{}. &{} -\frac{1-\gamma }{2} &{}. &{}. \\ . &{} -\frac{1-\gamma }{2} &{}. &{}. &{}. &{}. &{}. &{}. &{} \gamma w^* \\ . &{}. &{} \gamma w &{}. &{} 1-\gamma &{}. &{}. &{}. &{}. \\ . &{}. &{}. &{}. &{}. &{} \gamma &{}. &{} -\frac{1-\gamma }{2} &{}. \\ . &{}. &{} -\frac{1-\gamma }{2} &{}. &{}. &{}. &{} \gamma &{}. &{}. \\ \gamma w^* &{}. &{}. &{}. &{}. &{} -\frac{1-\gamma }{2} &{}. &{}. &{}. \\ . &{}. &{}. &{} \gamma w &{}. &{}. &{}. &{}. &{} 1-\gamma \\ \end{array} \right) , \end{aligned}$$

where, to make the formula more transparent, we replaces zeros by dots. It was proved^24^ that for each $\gamma \in (0,1)$, $W_{\gamma }$ is an optimal entanglement witness. However, being an optimal EW, it does not satisfy SPA conjecture. Indeed, it turns out^24^ that SPA of $W_\gamma$ defines an entangled state for some range of $\gamma$, specifically, for $\gamma \in (0.7,1)$. Consider now a mirrored operator

28 $$\begin{aligned} W_\gamma ^\mu =\mu \; {\mathbb {I}}_3 \otimes {\mathbb {I}}_3-W_\gamma . \end{aligned}$$

Clearly $\mu$ depends upon $\gamma$. It turns out that for $\gamma \ge 0.7$, one has $\mu =\frac{\gamma +1}{2}$.

Interestingly, both $W_\gamma$ and $W_\gamma ^\mu$ can detect the Bell states. However, we have not been successful to construct a bound entangled state which will be detected by $W_\gamma ^\mu$. We have tried all magic simplex states^31–33^, i.e. all possible convex combinations of a complete set of Bell states. Recently, the authors in Refs.^34–36^ have shown that with a probability of success of $95\%$, one can solve the separability problem for that huge family of states i.e. for Bell diagonal qutrit states with positive partial transposition. However, none of those states can be detected by the witness under our investigation. Summing up, our usual methods to find a PPT-entangled state, which is detected by $W_\gamma ^\mu$, have failed.

## Mirroring non-decomposable entanglement witnesses

In this Section, we analyze our conjecture for non-decomposable EWs. Again, we do not provide the proof but in what follows, we present several examples supporting the conjecture.

### EWs from unextendible product bases

Consider the well known EW proposed in Refs.^37,38^: if $|a_k \otimes b_k\rangle \in {\mathcal {H}}_A \otimes {\mathcal {H}}_B$ defines an unextendible product basis, i.e. an incomplete orthogonal product basis in ${\mathcal {H}}_A \otimes {\mathcal {H}}_B$ whose complementary subspace contains no product vector, then the following operator

29 $$\begin{aligned} W = \sum _k |a_k \otimes b_k\rangle \langle a_k \otimes b_k| - \epsilon _- \mathbbm {1}_{AB}, \end{aligned}$$

with

30 $$\begin{aligned} \epsilon _-:= \inf _{|a \otimes b\rangle } \, \sum _k |\langle a_k \otimes b_k| a \otimes b\rangle |^2, \end{aligned}$$

defines a non-decomposable EW. One finds for the mirrored operator

31 $$\begin{aligned} W_{\textrm{M}} = \epsilon _+ \mathbbm {1}_{AB} - \sum _k |a_k \otimes b_k\rangle \langle a_k \otimes b_k|, \end{aligned}$$

with

32 $$\begin{aligned} \epsilon _+:= \sup _{|a \otimes b\rangle } \, \sum _k |\langle a_k \otimes b_k| a \otimes b_k\rangle |^2 = 1. \end{aligned}$$

Hence, the mirrored operator is a projection onto a subspace orthogonal to $\textrm{span} \,\{|a_k \otimes b_k\rangle \}$. After an appropriate normalization, $W_{\textrm{M}}$ defines a PPT state and

33 $$\begin{aligned} \textrm{Tr}(W W_{\textrm{M}}) = - \epsilon _- \textrm{Tr} W_{\textrm{M}} < 0, \end{aligned}$$

that is, $W_{\textrm{M}}$ is a PPT-entangled operator detected by *W*^38^.

### Choi EW and its generalization in ${\mathbb {C}}^3 \otimes {\mathbb {C}}^3$

As a second example, let us consider a family of EWs in ${\mathbb {C}}^3 \otimes {\mathbb {C}}^3$ defined by^39^

34 $$\begin{aligned} W[a,b,c]= & {} \sum _{i=0}^2 \Big [\, a\, |ii\rangle \langle ii| + b\, |i,i+1\rangle \langle i,i+1| \nonumber \\{} & {} \quad + c\, |i,i+2\rangle \langle i,i+2|\, \Big ] - \sum _{i\ne j=0}^2 |ii\rangle \langle jj|, \end{aligned}$$

with $a,b,c\ge 0$ satisfying $a+b+c\ge 2$ and if $a \le 1$, then additionally $bc\ge (1-a)^2$. This family provides a generalization to the well known Choi witness corresponding to *W*[1, 1, 0] or *W*[1, 0, 1]^40,41^. Choi witness was proved to be extremal^42,43^ and hence also optimal. Interestingly, being optimal it does not have a spanning property^21,44^. A subclass of *W*[*a*, *b*, *c*] defined by^45^ (cf. also^46^)

$$\begin{aligned} a+b+c=2 \, \ \ \ a^2+b^2+c^2=2, \end{aligned}$$

was proved to be optimal if and only if $a \in [0,1]$, and extremal if and only if $a \in (0,1]$. Moreover, *W*[*a*, *b*, *c*] is decomposable only if $b=c=1$.

#### Proposition 4

*The mirrored operator to* (34) *is*

- *positive if* $$a \in [0,1/3]$$,
- *decomposable EW if* $a \in (1/3,4/3]$.

#### Proof

**Figure 3:**
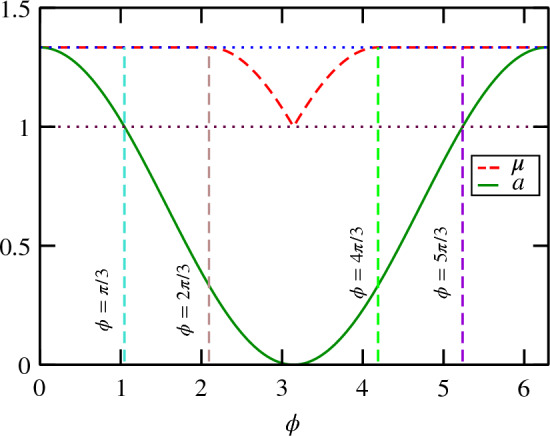
The plot of $\mu =\mu (\phi )$ and $a=a(\phi )$.

Let us use the following convenient parameterization^45,46^

35 $$\begin{aligned} a= & {} \frac{2}{3} ( 1 + \cos \phi ),\nonumber \\ b= & {} \frac{1}{3} ( 2 - \cos \phi - \sqrt{3} \sin \phi ),\nonumber \\ c= & {} \frac{1}{3} ( 2 - \cos \phi + \sqrt{3} \sin \phi ), \end{aligned}$$

that is, one has a 1-parameter family of EWs $W(\phi )$ for $\phi \in [0,2\pi )$. $W(\pi /3)$ and $W(5\pi /3)$ correspond to a pair of Choi witnesses and $W(\pi )$ corresponds to EW defined via the reduction map $R_3$. $W(\phi )$ is optimal iff $\phi \in [\pi /3,5\pi /3]$. One easily finds for $\mu (\phi )$ (cf. the Fig. 3)

36 $$\begin{aligned} \mu (\phi ) = \left\{ \begin{array}{ll} 4/3 &{}; \ \ \phi \in [0,2\pi /3] \cup [4 \pi /3,2\pi ) \\ c(\phi ) &{}; \ \ \phi \in [2\pi /3,\pi ] \\ b(\phi ) &{}; \ \ \phi \in [\pi ,4 \pi /3] \end{array} \right. \end{aligned}$$

such that the mirrored operator

37 $$\begin{aligned} W_{\textrm{M}}(\phi ) = \mu (\phi ) \mathbbm {1}_3 \otimes \mathbbm {1}_3 - W(\phi ), \end{aligned}$$

is block-positive. Note, that $b(\pi )=c(\pi )=1$. Now, for $\phi \in [0,2\pi /3] \cup [4 \pi /3,2\pi )$ one has

38 $$\begin{aligned} W_{\textrm{M}}(\phi ) = \frac{4}{3} \, \mathbbm {1}_3 \otimes \mathbbm {1}_3 - W(\phi ) = 3 \left( \frac{4}{3} -a \right) P^+_3 + B^\Gamma (\phi ), \end{aligned}$$

with

39 $$\begin{aligned} B(\phi )= & {} \left( \frac{4}{3} - b\right) \sum _{i=0}^2 |i\rangle \langle i|\otimes |i+1\rangle \langle i+1| \nonumber \\{} & {} \quad + \left( \frac{4}{3} - c\right) \sum _{i=0}^2 |i\rangle \langle i|\otimes |i+2\rangle \langle i+2| \nonumber \\{} & {} \quad + \left( a-\frac{1}{3}\right) \sum _{i\ne j=0}^2 |i\rangle \langle j|\otimes |j\rangle \langle i|. \end{aligned}$$

Indeed, for $\phi \in [0,2\pi /3] \cup [4 \pi /3,2\pi )$ one has $a,b,c \le 4/3$. Therefore, the first part of Eq. (38) is positive. We now will show that $B(\phi )\ge 0$. Note that the positivity of $B(\phi )$ is equivalent to positivity of the following $2\times 2$ submatrix

40 $$\begin{aligned} \left( \begin{array}{cc} 4/3 - b &{} a - 1/3 \\ a - 1/3 &{} 4/3 - c \end{array} \right) . \end{aligned}$$

Simple calculation shows that the determinant of this submatrix equals to 0, which proves that $B(\phi ) \ge 0$ and hence $W_{\textrm{M}}(a,b,c)$ is decomposable.

Now, if $\phi \in (2\pi /3,\pi ]$, we can express $W_{\textrm{M}}$ in the following form

41 $$\begin{aligned} W_{\textrm{M}}(\phi )= & {} (c-a) \sum _{i=0}^2 |i\rangle \langle i|\otimes |i\rangle \langle i| \nonumber \\{} & {} \quad + \left( c - b\right) \sum _{i=0}^2 |i\rangle \langle i|\otimes |i+1\rangle \langle i+1| \nonumber \\{} & {} \quad + \sum _{i\ne j=0}^2 |i\rangle \langle j|\otimes |i\rangle \langle j|, \end{aligned}$$

where

$$\begin{aligned} c-a = \frac{1}{3}(\sqrt{3} \sin \phi - 3 \cos \phi ) \ge 0, \ \ \ \ c-b = \frac{2}{\sqrt{3}}\, \sin \phi \ge 0. \end{aligned}$$

This proves that $W_{\textrm{M}}(\phi )\ge 0$ in $\phi \in (2\pi /3,\pi ]$. Similar analysis shows that $W_{\textrm{M}}(\phi ) \ge 0$ for $\phi \in [\pi ,4 \pi /3]$. We summarize our finding in Table 1. $\square$

#### Remark 2

Note that if $W_{\textrm{M}}(\phi ) \ge 0$, i.e. $\phi \in [2\pi /3,4\pi /3]$, then $\textrm{tr}[W_{\textrm{M}}(\phi )W(\phi )]<0$, i.e. $\rho = W_{\textrm{M}}(\phi )/\textrm{Tr}W_{\textrm{M}}(\phi )$ defines a PPT entangled state. Indeed, one has

42 $$\begin{aligned} \textrm{tr}[W_{\textrm{M}}(\phi )W(\phi )] = \mu (\phi ) \textrm{tr}W(\phi ) - \textrm{tr}[W(\phi )W(\phi )]. \end{aligned}$$

Note, that $\textrm{tr}W(\phi ) = 3(a+b+c)=6$ and $\textrm{tr}[W(\phi )W(\phi )] = 12$, and hence

43 $$\begin{aligned} \textrm{tr}[W_{\textrm{M}}(\phi )W(\phi )] = 6(\mu (\phi )-2) \end{aligned}$$

is always negative due to $\mu (\phi ) \le 4/3$.

**Table 1:** The regions of decomposability (D) or non-decomposability (ND) or positive operator (PO) for different values of $\phi$ in the scenario of entanglement witnesses $W(\phi )$ and their corresponding mirrored ones $W_{\textrm{M}}(\phi )$ in ${\mathbb {C}}^3 \otimes {\mathbb {C}}^3$.

$\phi$	Optimality of *W*	ND of *W*	$\mu$	${W_{\textrm{M}}}$
$[\pi /3,2 \pi /3]$	✓	✓	4/3	D
$[2 \pi /3, \pi )$	✓	✓	$c(\phi )$	PO
$\pi$	✓	✗	4/3	PO
$(\pi , 4 \pi /3]$	✓	✓	$b(\phi )$	PO
$[4 \pi /3, 5 \pi /3]$	✓	✓	4/3	D

### Mirrored pairs in ${\mathbb {C}}^4 \otimes {\mathbb {C}}^4$

Let us consider now a family of EWs being a generalization of a family *W*[*a*, *b*, *c*] in ${\mathbb {C}}^4 \otimes {\mathbb {C}}^4$^47^

44 $$\begin{aligned} W[a,b,c,d]= & {} \sum _{i=0}^3 \Big [\, a\, |ii\rangle \langle ii| + b\, |i,i+1\rangle \langle i,i+1| \nonumber \\{} & {} \quad + c\, |i,i+2\rangle \langle i,i+2| + d\, |i,i+3\rangle \langle i,i+3|\, \Big ] \nonumber \\{} & {} \quad - \sum _{i\ne j=0}^3 |ii\rangle \langle jj|, \end{aligned}$$

with $$a,b,c,d\ge 0$$ satisfying

45 $$\begin{aligned} a+b+c+d= & {} a^2+b^2+c^2+d^2 = 3, \end{aligned}$$

46 $$\begin{aligned} ac+bd= & {} 1, \ \ \ \ (a+c)(b+d) = 2. \end{aligned}$$

There are two solutions to the above set of equations^47^: class I is characterized by (45) together with

47 $$\begin{aligned} a+c=2 \, \ \ b+d=1, \end{aligned}$$

whereas class II is characterized by (45) together with

48 $$\begin{aligned} a+c=1 \, \ \ b+d=2. \end{aligned}$$

Interestingly, it is shown^47^ that EWs from class I are not optimal, whereas those from class II are optimal.

#### Example 3

Consider a Choi-like EWs *W*[1, 1, 1, 0]. Contrary to *W*[1, 1, 0] in ${\mathbb {C}}^3 \otimes {\mathbb {C}}^3$, it is not optimal. One easily finds the corresponding mirrored operator

49 $$\begin{aligned} W_{\textrm{M}}[1,1,1,0] = \frac{4}{3}\, \mathbbm {1}_4 \otimes \mathbbm {1}_4 - W[1,1,1,0]. \end{aligned}$$

It turns out that the mirrored operator $W_{\textrm{M}}[1,1,1,0]$ defines a non-decomposable EW. Indeed, by considering the following (unnormalized) state

50 $$\begin{aligned} \rho _x= & {} \sum _{i=0}^3 \Big [\, 3\, |ii\rangle \langle ii| + x\, |i,i+1\rangle \langle i,i+1| + \, |i,i+2\rangle \langle i,i+2| \nonumber \\{} & {} \quad + \frac{1}{x}\, |i,i+3\rangle \langle i,i+3|\, \Big ] - \sum _{i\ne j=0}^3 |ii\rangle \langle jj|\, \end{aligned}$$

where $x > 0$, it is easy to check that $\rho _x$ is PPT. Hence, we obtain

51 $$\begin{aligned} \textrm{tr}(W_{\textrm{M}}[1,1,1,0]\, \rho _x) = \frac{4}{3x}(x^2-5x +4). \end{aligned}$$

Clearly, $\textrm{tr}(W_{\textrm{M}}[1,1,1,0]\, \rho _x) < 0$ if and only if $x \in (1,4)$. This proves that $W_{\textrm{M}}[1,1,1,0]$ detects a PPT-entangled state $\rho _x$ and therefore, it is a non-decomposable EW. It is evident that in this case we have a pair of mirrored non-decomposable EWs $( W[1,1,1,0], W_{\textrm{M}}[1,1,1,0])$. This example shows that if one relaxes the requirement of optimality, then the mirrored operator might be non-decomposable EW as well.

Similar to the witnesses *W*[*a*, *b*, *c*] in ${\mathbb {C}}^3 \otimes {\mathbb {C}}^3$, *W*[*a*, *b*, *c*, *d*] can be parameterized as follows:

52 $$\begin{aligned} \text{ class } \text{ I }:~~~a= & {} \frac{1}{2} (2-\sin \theta ) \, \ b = \frac{1}{2} (1+\cos \theta ) \, \nonumber \\ \ c= & {} 2-a \, ~~~~~~~~~~ d = 1- b \, \end{aligned}$$

and

53 $$\begin{aligned} \text{ class } \text{ II }:~~~a= & {} \frac{1}{2} (1+\cos \theta ) \, \ b = \frac{1}{2} (2-\sin \theta ) \, \nonumber \\ \ c= & {} 1-a\, ~~~~~~~~~~ d = 2-b \, \end{aligned}$$

with $\theta \in [0,\pi ]$. We use the notations $W_I(\theta )$ and $W_{II}(\theta )$ for *W*[*a*, *b*, *c*, *d*] in the first and second classes, respectively. In particular, $W_I(0) = W[1,1,1,0]$ and $W_I(\pi ) = W[1,0,1,1]$ are Choi-like EWs, $W_I(\pi /2) = W[1/2,1/2,3/2,1/2]$ is the only decomposable EW in the class I. Similarly, $W_{II}(\pi ) = W[0,1,1,1]$ corresponds to the reduction map $R_4$, whereas $W_{II}(0) = W[1,1,0,1]$ is the second decomposable EW in the class II (cf.^47^).

For $\theta \in (0,\pi )$, the class II consists of non-decomposable EWs. One finds for the mirrored operators

54 $$\begin{aligned} W^{\textrm{M}}_{II}(\theta ) = \mu (\theta ) \mathbbm {1}_4 \otimes \mathbbm {1}_4 - W_{II}(\theta ), \end{aligned}$$

with

55 $$\begin{aligned} \mu (\theta ) = \left\{ \begin{array}{ll} 3/2 &{}; \ \ \theta \in (0,\pi /2) \\ d(\theta ) &{}; \ \ \theta \in (\pi /2,\pi ) \end{array} \right. \end{aligned}$$

#### Proposition 5

*The mirrored operator to* $W_{II}(\theta )$ *is*

- *decomposable EW if* $\theta \in (0,\pi /2)$,
- *positive if* $$\theta \in (\pi /2,\pi )$$.

The proof is very similar to that of Proposition 4 (see Supplementary Material).

#### Remark 3

Note that if $W^{\textrm{M}}_{II}(\theta ) \ge 0$, i.e. $\theta \in (\pi /2,\pi )$, then $\textrm{tr}[W_{II}(\theta )W^{\textrm{M}}_{II}(\theta )]<0$, that is, $\rho = W^{\textrm{M}}_{II}(\theta )/\textrm{Tr}W^{\textrm{M}}_{II}(\theta )$ defines a PPT enatgled state. Indeed, one has

56 $$\begin{aligned} \textrm{tr}[W^{\textrm{M}}_{II}(\theta )W_{II}(\theta )] = \mu (\theta ) \textrm{tr}W_{II}(\theta ) - \textrm{tr}[W_{II}(\theta )W_{II}(\theta )]. \nonumber \\ \end{aligned}$$

Note, that $\textrm{tr}W_{II}(\theta ) = 4(a+b+c+d)=12$ and $\textrm{tr}[W_{II}(\theta )W_{II}(\theta )] = 24$, and hence

57 $$\begin{aligned} \textrm{tr}[W_{II}(\theta )W^{\textrm{M}}_{II}(\theta )] = 12(\mu (\theta )-2) \end{aligned}$$

is always negative due to $\mu (\theta ) \le 3/2$.

Now, the class I contains non-optimal EWs. In Ref.^47^ by following the paper^12^, an optimization procedure was performed leading to an optimal EW defined via

58 $$\begin{aligned} {\tilde{W}}_I(\theta ) = W_I(\theta ) - 2 P, \end{aligned}$$

where $P=|\Psi \rangle \langle \Psi |$ is a rank-1 projector onto the maximally entangled state in ${\mathbb {C}}^4 \otimes {\mathbb {C}}^4$ with $|\Psi \rangle = \frac{1}{2} \sum _{j=0}^3 (-1)^{j} |j \otimes j\rangle$.

#### Proposition 6

*The mirrored operator to* ${\tilde{W}}_{I}(\theta )$ *for* $\theta \in [0,\pi ] - \{\pi /2\}$

59 $$\begin{aligned} {\tilde{W}}^{\textrm{M}}_{I}(\theta ) = \frac{3}{2} \mathbbm {1}_4 \otimes \mathbbm {1}_4 - {\tilde{W}}_{I}(\theta ), \end{aligned}$$

*is a decomposable EW*.

The proof is very similar to that of Proposition 4 (see Supplementary Material).

### A class of non-decomposable Breuer–Hall maps

In Refs.^48,49^, Breuer and Hall have generalized the reduction map by the following class of positive maps $\Phi : M_{2n}({\mathbb {C}}) \rightarrow M_{2n}({\mathbb {C}})$ such that

60 $$\begin{aligned} \Phi _{\textrm{BH}}(X)=R_{2n}(X)-UX^TU^\dagger , \end{aligned}$$

where *U* is an arbitrary antisymmetric unitary matrix in $M_{2n}({\mathbb {C}})$. It was shown that this map is non-decomposable^48,49^ and optimal^48^, even nd-optimal^12,48^. The mirrored positive map

61 $$\begin{aligned} \Phi _{\textrm{BH}}^{\textrm{M}}(X)= \mathbbm {1}_{2n} \textrm{tr}X - \Phi _{\textrm{BH}}(X) = X + UX^TU^\dagger , \end{aligned}$$

which is evidently decomposable being a sum of an identity and completely co-positive map $UX^TU^\dagger$.

### A class of non-decomposable maps in $M_n({\mathbb {C}}) \otimes M_n({\mathbb {C}})$

Let $\varepsilon : M_n({\mathbb {C}}) \rightarrow M_n({\mathbb {C}})$ be the canonical projection of $M_n({\mathbb {C}})$ to the diagonal part

62 $$\begin{aligned} \varepsilon (X)=\sum _{i=0}^{n-1} \langle i|X| {i}\rangle |i\rangle \langle i|. \end{aligned}$$

Let *S* be a permutation defined by

63 $$\begin{aligned} S |i\rangle =|i+1\rangle , \ \ \ \ (\text{ mod }~n), \end{aligned}$$

for $i=0,1,\ldots , n-1$. The following maps $\tau _{n,k} : M_n({\mathbb {C}}) \rightarrow M_n({\mathbb {C}})$

64 $$\begin{aligned} \tau _{n,k}(X)=(n-k) \varepsilon (X)+\sum _{i=1}^{k} \varepsilon \big (S^i X S^{\dagger i}\big )-X, \end{aligned}$$

for $k=0,1,\ldots ,n-1$, were proved to be positive and non-decomposable if $k<n-1$^50–55^. Actually, $\tau _{n,n-1} = R_n$ (reduction map), and $\tau _{3,1}$ is a Choi map in $M_3({\mathbb {C}})$.

Now, the mirrored map is defined via

65 $$\begin{aligned} \tau ^{\textrm{M}}_{n,k}(X) = \mu _{n,k}\, \mathbbm {1}_n \textrm{tr}X - \tau _{n,k}(X). \end{aligned}$$

Let us denote the greatest common divisor of *n* and *k* by $\textrm{gcd}(n,k)$. In a recent paper^26^, the authors have shown that if $\textrm{gcd}(n,k)=1$, then $\tau _{n,k}$ is optimal. In particular, $\textrm{gcd}(n,1)=1$ and we find that

66 $$\begin{aligned} \mu _{n,1} = \left\{ \begin{array}{ll} \frac{4}{3} ;\ {} &{} \ n=3, \\ n-2; \ &{} \ n \ge 4. \end{array} \right. \end{aligned}$$

Similarly, for odd *n*, one has $\textrm{gcd}(n,n-2)=1$ and we obtain

67 $$\begin{aligned} \mu _{n,n-2} = \left\{ \begin{array}{ll} \frac{4}{3}; \ {} &{} \ n=3, \\ \frac{3}{2}; \ {} &{} \ n \ge 4. \end{array} \right. \end{aligned}$$

#### Proposition 7

*The mirrored maps* $\tau ^{\textrm{M}}_{n,1}$ *and* $\tau ^{\textrm{M}}_{n,n-2}$ *are decomposable*.

#### Proof

Due to Choi-Jamiołkowski isomorphism, the EW corresponding to the map $\tau _{n,k}$ can be expressed as

68 $$\begin{aligned} W_{n,k}= & {} \sum _{i,j=0}^{n-1} |i\rangle \langle j| \otimes \tau _{n,k}(|i\rangle \langle j|) \nonumber \\= & {} \sum _{i=0}^{n-1}\Big [ (n-k-1)|ii\rangle \langle ii|+ \sum _{\ell =1}^k |i,i+\ell \rangle \langle i,i+\ell | \Big ] \nonumber \\- & {} \sum _{i\ne j} |ii\rangle \langle jj|. \end{aligned}$$

Therefore, for $n \ge 4$, one can easily rite the corresponding mirrored operator

69 $$\begin{aligned} W^{\textrm{M}}_{{n,1}}= & {} \sum _{i=0}^{n-1} \Big [ (n-3) |i,i+1\rangle \langle i, i+1| \nonumber \\ {}+ & {} (n-2) \sum _{\ell =2}^{n-1} |i,i+\ell \rangle \langle i,i+\ell | \Big ] + \sum _{i \ne j=0}^{n-1} |ii \rangle \langle jj |. \end{aligned}$$

Note, that $W^{\textrm{M}}_{{n,1}} = B_{n,1}^\Gamma$, with $B_{n,1}^\Gamma \ge 0$. Actually, positivity of $B_{n,1}$ is equivalent to the positivity of the following $2\times 2$ submatrix

70 $$\begin{aligned} \left( \begin{array}{cc} n-3 &{} 1 \\ 1 &{} n-2 \end{array} \right) , \end{aligned}$$

which is evidently positive for $n\ge 4$. This implies that $W^{\textrm{M}}_{\tau _{n,1}}$ is decomposable for $n \ge 4$.

Now, for the odd *n*, $\tau _{n,n-2}$ produces again the Choi map for $n=3$ that we discussed above. For $n>3$, the corresponding mirrored EW for $\mu =3/2$ can be expressed as follows

71 $$\begin{aligned} W^M_{{n,n-2}}= & {} \frac{1}{2} \sum _{i=0}^{n-1} \Big [ \sum _{\ell =0}^{n-2} |i,i+\ell \rangle \langle i,i+\ell | \nonumber \\+ & {} \, 3|i,i+n-1\rangle \langle i,i+n-1| \Big ] + \sum _{i \ne j=0}^{n-1} |ii \rangle \langle jj |. \end{aligned}$$

Moreover, $W^M_{\tau _{n,n-2}}$ can be written as $W^M_{{n,n-2}}=A_{n,n-2}+B_{n,n-2}^\Gamma$, where

72 $$\begin{aligned} A_{n,n-2} =\frac{1}{2} \sum _{i \ne j=0}^{n-1} \Big [|ii\rangle \langle ii|+ |ii\rangle \langle jj| \Big ] >0, \end{aligned}$$

and

73 $$\begin{aligned} B_{n,n-2}= & {} \frac{1}{2} \Big [ \sum _{i=0}^{n-1} \Big ( \sum _{\ell =1}^{n-2} |i,i+\ell \rangle \langle i, i+\ell | \nonumber \\{} & {} \quad + 3|i,i+n-1\rangle \langle i,i+n-1| \Big ) + \sum _{i \ne j=0}^{n-1} |ij \rangle \langle ji| \Big ]. \end{aligned}$$

It is easy to see that $B_{n,n-2}$ is positive as the positivity of $B_{n,n-2}$ is equivalent to the positivity of the following $2\times 2$ submatrix

74 $$\begin{aligned} \left( \begin{array}{cc} 1 &{} 1 \\ 1 &{} 3 \end{array} \right) . \end{aligned}$$

This shows that $W^M_{{n,n-2}}$ is decomposable for $n \ge 4$. $\square$

### A family of optimal non-decomposable witnesses in ${\mathbb {C}}^3 \otimes {\mathbb {C}}^3$ whose SPA is not separable

In this section, we consider a family of indecomposable entanglement witnesses proposed by Ha and Kye in Ref.^22^ whose SPAs are not separable. For non-negative real numbers *a*, *b*, *c* and $-\pi \le \theta \le \pi$, the form of the self-adjoint block matrix in ${\mathbb {C}}^3 \otimes {\mathbb {C}}^3$ is given by

75 $$\begin{aligned} W[a,b,c;\theta ]=\left( \begin{array}{ccc|ccc|ccc} a &{}. &{}. &{}. &{} -e^{i \theta } &{}. &{}. &{}. &{} -e^{-i \theta } \\ . &{} b &{}. &{}. &{}. &{}. &{}. &{}. &{}. \\ . &{}. &{} c &{}. &{}. &{}. &{}. &{}. &{}. \\ \hline . &{}. &{}. &{} c &{}. &{}. &{}. &{}. &{}. \\ -e^{-i \theta } &{}. &{}. &{}. &{} a &{}. &{}. &{}. &{} -e^{i \theta } \\ . &{}. &{}. &{}. &{}. &{} b &{}. &{}. &{}. \\ \hline . &{}. &{}. &{}. &{}. &{}. &{} b &{}. &{}. \\ . &{}. &{}. &{}. &{}. &{}. &{}. &{} c &{}. \\ -e^{i \theta } &{}. &{}. &{}. &{} -e^{-i \theta } &{}. &{}. &{}. &{} a \\ \end{array} \right) . \end{aligned}$$

Let $p_\theta =\max \{q_{(\theta -\frac{2}{3} \pi )},q_\theta ,q_{(\theta +\frac{2}{3} \pi )} \}$, where $q_\theta =e^{i \theta }+e^{-i\theta }$. One has $1 \le p_\theta \le 2$. Now, $W[a,b,c;\theta ] \ge 0$ iff $a \ge p_\theta$ and $W[a,b,c;\theta ]$ is is block-positive iff the following conditions hold

76 $$\begin{aligned} 1) \ \ a+b+c \ge p_\theta , \ \ \ \ 2) \ \ \text{ if } \ a\le 1, \ \ \text{ then } \ \ bc \ge (1-a)^2. \end{aligned}$$

The authors of^22^ analyzed two classes of EWs:

77 $$\begin{aligned} 2-p_\theta \le a<1, \ \ \ a+b+c=p_\theta , \ \ \ bc=(1-a)^2, \end{aligned}$$

and

78 $$\begin{aligned} 1 \le a<p_\theta ,\ \ \ a+b+c=p_\theta ,\ \ \ bc=0, \end{aligned}$$

For (77) one has $4/3 \le p_\theta < 1+1/\sqrt{2}$ and for (78) one has $1+1/\sqrt{2} \le p_\theta < 2$. Both classes consist of non-decomposable EWs. Moreover, class (77) has the bi-spanning property, whereas class (78) has the co-spanning property^22^. For the first class let us consider $p_\theta =4/3$, $a=2-p_\theta =2/3$, and $b=c=p_\theta -1=1/3$. Then the corresponding mirrored EW is $W'=\mu {\mathbb {I}}_3 \otimes {\mathbb {I}}_3-W[a,b,c;\theta ]$, where $\mu =1.097$. Let us observe that one can easily construct a PPT state

79 $$\begin{aligned} \rho _x{} & {} = \sum _{i=0}^2 \Big [ x |i,i\rangle \langle i,i| + |i+1,i+1\rangle \langle i+1,i+1| \nonumber \\{} & {} \quad + |i+2\rangle \langle i+2| + e^{i\theta } |i,i\rangle \langle i+1,i+1| \nonumber \\{} & {} \quad + e^{-i\theta }|i,i\rangle \langle i+2,i+2| \Big ], \end{aligned}$$

which is PPT if and only if

80 $$\begin{aligned} \left( \begin{array}{ccc} x &{} e^{i\theta } &{} e^{-i\theta } \\ e^{-i\theta } &{} x &{} e^{i\theta } \\ e^{i\theta } &{} e^{-i\theta } &{} x \end{array} \right) \ge 0, \end{aligned}$$

that is, $x \ge 1$ and $x^3 + 2 \cos (3\theta ) - 3 x \ge 0$ which for $p_\theta = 4/3$ implies $x \ge 1.896$. One finds

81 $$\begin{aligned} \textrm{Tr}(W[2/3,1/3,1/3,\theta ]\, \rho _x) = 2(x-2) < 0, \end{aligned}$$

for $x \in [1.896,2)$ which proves that $W[2/3,1/3,1/3,\theta ]$ is non-decomposable. Consider now the following state

82 $$\begin{aligned} \rho _y{} & {} = \sum _{i=0}^2 \Big [ y |i,i\rangle \langle i,i| + |i+1,i+1\rangle \langle i+1,i+1| \nonumber \\{} & {} \quad + |i+2\rangle \langle i+2| - e^{i\theta } |i,i\rangle \langle i+1,i+1| \nonumber \\{} & {} \quad - e^{-i\theta }|i,i\rangle \langle i+2,i+2| \Big ], \end{aligned}$$

which is PPT if and only if

83 $$\begin{aligned} \left( \begin{array}{ccc} y &{} -e^{i\theta } &{} -e^{-i\theta } \\ -e^{-i\theta } &{} y &{} -e^{i\theta } \\ -e^{i\theta } &{} -e^{-i\theta } &{} y \end{array} \right) \ge 0, \end{aligned}$$

that is, $y \ge 1$ and $xy^3 - 2 \cos (3\theta ) - 3 y \ge 0$ which, for $p_\theta = 4/3$, implies $y \ge 3/2$. One finds $\textrm{Tr}((\mu {\mathbb {I}}_3 \otimes {\mathbb {I}}_3 - W[2/3,1/3,1/3,\theta ])\, \rho _y) > 0.5185$ for $y \ge 3/2$. Clearly, it does not prove that the witness is decomposable. However, both the witness and the state $\rho _y$ have the same symmetry and it is natural to expect that a proper PPT state which detects non-decomposability of the witness belongs to the above family of states. Additionally, we performed numerical analysis and used all bound entangled states found in the magic simplex, i.e. Bell diagonal states, based on a grid approach (140,000 states)^34^ and based on a representative random sample which fills the volume of the bound entangled states within the magic simplex ^35^. Note that the classification of bound entangled states in the magic simplex is obtained with a very high success rate of $5\%$. Obviously, the given witness must not be sensitive to Bell diagonal states. Therefore, we used the sequentially constrained Monte Carlo method introduced in Ref.^56^ to sample from a random set of 100,000 states in this case 8007 bound entangled states detected by the realignment criterion. Each state was also optimized over local unitaries with the convenient composite parametrization introduced in Ref.^57^. None of these bound entangled states detects non-decomposability of $W'$.

For the second class (78) we consider $p_\theta = 1+1/\sqrt{2}$ and hence $a=1, b=p_\theta -1$, $c=0$, or $a=1$, $b=0$, $c=p_\theta -1$. Hence the corresponding mirrored EW is $W''=\mu \; {\mathbb {I}}_3 \otimes {\mathbb {I}}_3-W[a,b,c;\theta ]$, where $\mu '=1.21473$. A similar analysis as for the class (77) supports our conjecture. Numerical analysis shows that no bound entangled state is detected by this witness.

## Conclusions

As entanglement is generally a useful resource in quantum information theory, its verification has both fundamental and practical significance^58^. It is, however, generally challenging as its computational complexity lies in the NP-hard class. The problem is also connected to a long–standing open question about the classification of positive maps. In this paper, we have approached the problem by exploiting the convexity of separable states and EWs since optimal EWs define the set of separable states and non-decomposable EWs classify the set of PPT-entangled and free entangled states. PPT-entangled states are of particular interest related to numerous open questions in quantum information theory such as Bell inequalities, activation of entanglement, channel capacities, invariance under Lorentz boosts^59^, etc. Only limited knowledge is known about the structure of the PPT-entangled states in the Hilbert space and also only for low dimensions. We provide here a different view via the structure of connected entanglement witnesses.

Along with the convexity of quantum states and EWs, there has been the so-called SPA conjecture that addresses SPAs to optimal EWs are separable states. Counterexamples, however, exist. In our work, we have considered the framework of mirrored EW i.e. ‘twin’ of an EW such that both the EWs can detect the entangled states by realizing a single observable. A trade-off relation is observed between the EW and its mirrored ones, which we have presented as a conjecture in our paper. Our conjecture states that mirrored operators to optimal EWs are either quantum states or decomposable EWs, hence cannot detect the PPT-entangled states. In other words, there does not exist a mirrored pair of non-decomposable EWs such that at least one of them is optimal. Consequently, if our conjecture holds generally, then there is a relation between optimality of an EW and decomposability.

We have proved that mirrored EWs to extremal decomposable witnesses are positive semi-definite i.e., quantum states. In fact, for the extremal decomposable EWs, both our conjecture and the SPA one hold true. For non-decomposable EWs, several examples that support our conjecture are presented. In particular, those examples that disproved the SPA conjecture are considered: for all of the cases, their mirrored operators cannot detect PPT-entangled states. Let us reiterate that the assumption of optimality is essential: otherwise, one can immediately find examples of non-optimal and non-decomposable EWs such that their mirrored operators are also non-decomposable EWs.

We believe that our analysis unfolds a hidden structure of the set of entanglement witnesses, which brings us closer to the understanding of the separability problem. It should be stressed that the entire analysis can be generalized for the multipartite scenario^60^. In this case the picture is much more involved due to the fact that there is a whole hierarchy of *k*-separable states. If $W_k$ is an entanglement witness acting on ${\mathcal {H}}_1 \otimes \ldots \otimes {\mathcal {H}}_N$ which is positive on *k*-separable states, then one can define a family of mirrored EWs

84 $$\begin{aligned} W^M_{\ell }:= \mu _{k,\ell } \mathbbm {1}_1 \otimes {\cdot\cdot\cdot} \otimes \mathbbm {1}_N - W_k, \end{aligned}$$

such that $W_\ell ^M$ is positive on $\ell$-separable states. It would be interesting to analyze the properties of the mirrored pairs $(W_k,W^M_\ell )$. Another interesting point is to replace identity operator $\mathbbm {1}_1 \otimes \ldots \otimes \mathbbm {1}_N$ by arbitrary *N*-separable operator *X* acting on ${\mathcal {H}}_1 \otimes \ldots \otimes {\mathcal {H}}_N$ (cf.^61^). We postpone these issues for the future research.

## Supplementary Information

Supplementary Information.

## Data Availability

All data generated or analysed during this study are included in this published article [and its supplementary information files].

## Code availability

The results were computed by pencil or the use of commercial computer software as Mathematica.

## References

[1] Horodecki R, Horodecki P, Horodecki M, Horodecki K (2009). Quantum entanglement. Rev. Mod. Phys..

[2] Terhal BM (2000). Bell inequalities and the separability criterion. Phys. Lett. A.

[3] Gühne O, Tóth G (2009). Entanglement detection. Phys. Rep..

[4] Chruściński D, Sarbicki G (2014). Entanglement witnesses: Construction, analysis and classification. J. Phys. A..

[5] Kye S-H (2013). Facial structures for various notions of positivity and applications to the theory of entanglement. Rev. Math. Phys..

[6] Bera Anindita, Mal Shiladitya, Sen(De) A, Sen U (2018). Witnessing bipartite entanglement sequentially by multiple observers. Phys. Rev. A.

[7] Jamiołkowski A (1972). Linear transformations which preserve trace and positive semidefiniteness of operators. Rep. Math. Phys..

[8] Choi M-D (1975). Completely positive linear maps on complex matrices. Linear Algebra Appl..

[9] Paulsen V (2003). Completely Bounded Maps and Operator Algebras.

[10] Størmer E (2013). Positive Linear Maps of Operator Algebras, Springer Monographs in Mathematics.

[11] Bera, A., Scala, G., Sarbicki, G. & Chruściński, D. Generalizing Choi map in $$M_3$$ beyond circulant scenario. http://arxiv.org/abs/2212.03807.

[12] Lewenstein M, Kraus B, Cirac JI, Horodecki P (2000). Optimization of entanglement witnesses. Phys. Rev. A.

[13] Horodecki P (2003). From limits of quantum operations to multicopy entanglement witnesses and state-spectrum estimation. Phys. Rev. A.

[14] Horodecki P, Ekert A (2002). Method for direct detection of quantum entanglement. Phys. Rev. Lett..

[15] Korbicz JK, Almeida ML, Bae J, Lewenstein M, Acin A (2008). Structural approximations to positive maps and entanglement breaking channels. Phys. Rev. A.

[16] Augusiak R, Bae J, Czekaj L, Lewenstein M (2011). On structural physical approximations and entanglement breaking maps. J. Phys. A..

[17] Augusiak R, Bae J, Tura J, Lewenstein M (2014). Checking the optimality of entanglement witnesses: An application to structural physical approximations. J. Phys. A..

[18] Shultz F (2016). The structural physical approximation conjecture. J. Math. Phys..

[19] Chruściński D, Pytel J, Sarbicki G (2009). Constructing new optimal entanglement witnesses. Phys. Rev. A.

[20] Chruściński D, Pytel J (2011). Optimal entanglement witnesses from generalized reduction and Robertson maps. J. Phys. A.

[21] Ha K-C, Kye S-H (2011). One-parameter family of indecomposable optimal entanglement witnesses arising from generalized Choi maps. Phys. Rev. A.

[22] Ha K-C, Kye S-H (2012). The structural physical approximations and optimal entanglement witnesses. J. Math. Phys..

[23] Størmer E (2013). Separable states and the structural physical approximation of a positive map. J. Funct. Anal..

[24] Chruściński D, Sarbicki G (2014). Disproving the conjecture on the structural physical approximation to optimal decomposable entanglement witnesses. J. Phys. A.

[25] Bae J, Chruściński D, Hiesmayr BC (2020). Mirrored entanglement witnesses. NPJ Quant. Inf..

[26] Bera A, Sarbicki G, Chruściński D (2023). A class of optimal positive maps in $$M_n$$. Linear Algebra Appl..

[27] Parthasarathy KR (2004). On the maximal dimension of a completely entangled subspace for finite level quantum systems. Proc. Math. Sci..

[28] Cubitt T, Montanaro A, Winter A (2008). On the dimension of subspaces with bounded Schmidt rank. J. Math. Phys..

[29] Bae J, Bera A, Chruściński D, Hiesmayr BC, McNulty D (2022). How many mutually unbiased bases are needed to detect bound entangled states. J. Phys. A.

[30] Lewenstein M, Kraus B, Horodecki P, Cirac JI (2001). Characterization of separable states and entanglement witnesses. Phys. Rev. A.

[31] Baumgartner B, Hiesmayr BC, Narnhofer H (2006). State space for two qutrits has a phase space structure in its core. Phys. Rev. A.

[32] Baumgartner B, Hiesmayr BC, Narnhofer H (2007). Special simplex in the state space for entangled qudits. J. Phys. A.

[33] Baumgartner B, Hiesmayr BC, Narnhofer H (2008). The geometry of bipartite qutrits including bound entanglement. Phys. Lett. A.

[34] Hiesmayr BC (2021). Free versus bound entanglement: Machine learning tackling a NP-hard problem. Sci. Rep..

[35] Popp Ch, Hiesmayr BC (2022). Almost complete solution for the NP-hard separability problem of Bell diagonal qutrits. Sci. Rep..

[36] Popp, C. & Hiesmayr, B. C. *Bound Entanglement of Bell Diagonal Pairs of Qutrits and Ququarts: A Comparison*. http://arxiv.org/abs/2209.15267. 10.1038/s41598-023-29211-w PMC9899246 36739347

[37] Bennett CH, DiVincenzo DP, Mor T, Shor PW, Smolin JA, Terhal BM (1999). Unextendible product bases and bound entanglement. Phys. Rev. Lett..

[38] Terhal BM (2001). A Family of indecomposable positive linear maps based on entangled quantum states. Linear Algebra Appl..

[39] Cho SJ, Kye S-H, Lee SG (1992). Generalized Choi maps in 3-dimensional matrix algebra. Linear Algebra Appl..

[40] Choi MD (1975). Positive semidefinite biquadratic forms. Linear Algebra Appl..

[41] Choi MD (1982). Positive linear maps. Proc. Symp. Pure Math..

[42] Choi MD, Lam TY (1977). Extremal positive semidefinite forms. Math. Ann..

[43] Ha K-C (2013). Notes on extremality of the Choi map. Linear Algebra Appl..

[44] Chruściński D, Sarbicki G (2013). Optimal entanglement witnesses for two qutrits. Open Syst. Inf. Dyn..

[45] Kossakowski A (2003). A class of linear positive maps in matrix algebras. Open Syst. Inf. Dyn..

[46] Chruściński D, Wudarski FA (2011). Geometry of entanglement witnesses for two qutrits. Open Syst. Inf. Dyn..

[47] Bera A, Wudarski FA, Sarbicki G, Chruściński D (2022). Class of Bell-diagonal entanglement witnesses in $${\mathbb{C} }^4 \otimes {\mathbb{C} }^4$$: Optimization and the spanning property. Phys. Rev. A.

[48] Breuer H-P (2006). Optimal entanglement criterion for mixed quantum states. Phys. Rev. Lett..

[49] Hall W (2006). A new criterion for indecomposability of positive maps. J. Phys. A.

[50] Tanahashi K, Tomiyama J (1988). Indecomposable positive maps in matrix algebras. Can. Math. Bull..

[51] Osaka H (1991). Indecomposable positive maps in low dimensional matrix algebra. Linear Algebra Appl..

[52] Osaka H (1993). A series of absolutely indecomposable positive maps in matrix algebras. Linear Algebra Appl..

[53] Ando, T. Positivity of certain maps, Seminar Notes, 1985 (cited in [31]).

[54] Ha K-C (1998). Atomic positive linear maps in matrix algebras. Publ. RIMS.

[55] Yamagami S (1993). Cyclic inequalities. Proc. Am. Math. Soc..

[56] Li, W., Han, R., Shang, J., Ng, H. K. & Englert, B.-G. *Sequentially Constrained Monte Carlo Sampler for Quantum States*. http://arxiv.org/abs/2109.14215.

[57] Spengler C, Huber M, Hiesmayr BC (2012). Composite parameterization and Haar measure for all unitary and special unitary groups. J. Math. Phys..

[58] Bera A, Kumar A, Rakshit D, Prabhu R, Sen A, Sen U (2016). Information complementarity in multipartite quantum states and security in cryptography. Phys. Rev. A.

[59] Caban, P. & Hiesmayr, B. C. Is bound entanglement Lorentz invariant? http://arxiv.org/abs/2212.01286. 10.1038/s41598-023-38217-3 PMC10336123 37433840

[60] Zhou Y, Zhao Q, Yuan X, Ma X (2019). Detecting multipartite entanglement structure with minimal resources. NPJ Quant. Inf..

[61] Zhou Y (2020). Entanglement detection under coherent noise: Greenberger–Horne–Zeilinger-like states. Phys. Rev. A.

