# Supplementary material for: On the structure of mirrored operators obtained from optimal entanglement witnesses
Source: Sci Rep. 2023 Jul 3;13:10733. doi: 10.1038/s41598-023-37771-0 (PMC10318014; doi:10.1038/s41598-023-37771-0)
Supplement: Supplementary file 1 — Supplementary Information. [file 41598_2023_37771_MOESM1_ESM.pdf]

begindocument/before

SUPPLEMENTARY INFORMATION: On the structure of mirrored operators obtained from optimal entanglement witnesses

Anindita Bera,[1,*] Joonwoo Bae,[2] Beatrix C. Hiesmayr,[3] and Dariusz Chruściński[1]

[1]*Institute of Physics, Faculty of Physics, Astronomy and Informatics, Nicolaus Copernicus University, Grudziądzka 5/7, 87–100 Toruń, Poland*

[2]*School of Electrical Engineering, Korea Advanced Institute of Science and Technology (KAIST), 291 Daehak-ro, Yuseong-gu, Daejeon 34141, Republic of Korea*

[3]*University of Vienna, Faculty of Physics, Währingerstrasse 17, 1090 Vienna, Austria*

1. Supplementary notes

Proof of Proposition 5:

For $\theta \in (0, \pi/2)$, the corresponding mirrored operator of $W_{II}(\theta)$ can be written as

$$W_{II}^{\rm M}(\theta) = \frac{3}{2}\, \mathbb{1}_4 \otimes \mathbb{1}_4 - W_{II}(\theta) = 4\left(\frac{3}{2} - a\right) P_4^+ + B_1^\Gamma(\theta), \tag{1}$$

with

$$\begin{aligned} B_1(\theta) &= \left(\frac{3}{2} - b\right)\sum_{i=0}^{3} |i\rangle\langle i| \otimes |i+1\rangle\langle i+1| + \left(\frac{3}{2} - c\right)\sum_{i=0}^{3} |i\rangle\langle i| \otimes |i+2\rangle\langle i+2| \\ &+ \left(\frac{3}{2} - d\right)\sum_{i=0}^{3} |i\rangle\langle i| \otimes |i+3\rangle\langle i+3| + \left(a - \frac{1}{2}\right)\sum_{i\neq j=0}^{3} |i\rangle\langle j| \otimes |j\rangle\langle i|. \end{aligned} \tag{2}$$

Clearly, for $\theta \in (0, \pi/2)$, one has $\frac{1}{2} \leq a, b \leq 1,\ 0 \leq c \leq \frac{1}{2},\ 1 \leq d \leq \frac{3}{2}$. Hence, the first part of Eq. (1) is positive. Now we need to show that $B_1(\theta) \geq 0$. Note that the positivity of $B_1(\theta)$ is equivalent to the positivity of the following two 2×2 submatrices

$$A_1 = \begin{pmatrix} \frac{3}{2} - b & a - \frac{1}{2} \\ a - \frac{1}{2} & \frac{3}{2} - d \end{pmatrix} \quad \text{and} \quad A_2 = \begin{pmatrix} \frac{3}{2} - c & a - \frac{1}{2} \\ a - \frac{1}{2} & \frac{3}{2} - c \end{pmatrix}. \tag{3}$$

Simple calculation shows that $\det[A_1] = 0$ and $\det[A_2] = 1 + \cos\theta = 2a \geq 0$ in $\theta \in (0, \pi/2)$. This proves that $B_1(\theta) \geq 0$ and hence $W_{II}^{\rm M}(\theta)$ is decomposable.

Now, for $\theta \in (\pi/2, \pi)$, one can express $W_{II}^{\rm M}$ in the following way

$$\begin{aligned} W_{II}^{\rm M}(\theta) &= (d - a)\sum_{i=0}^{3} |i\rangle\langle i| \otimes |i\rangle\langle i| + (d - b)\sum_{i=0}^{3} |i\rangle\langle i| \otimes |i+1\rangle\langle i+1| \\ &+ (d - c)\sum_{i=0}^{3} |i\rangle\langle i| \otimes |i+2\rangle\langle i+2| + \sum_{i\neq j=0}^{3} |i\rangle\langle j| \otimes |i\rangle\langle j|. \end{aligned} \tag{4}$$

where

$$\begin{aligned} d - a &= \frac{1}{2}(\sin\theta - \cos\theta + 1) \geq 0, \quad d - b = \sin\theta \geq 0, \\ d - b &= \frac{1}{2}(\sin\theta + \cos\theta + 1) \geq 0. \end{aligned} \tag{5}$$

This proves that $W_{II}^{\rm M}(\theta) \geq 0$ in $\theta \in (\pi/2, \pi)$.

* anindita.bera@umk.pl

Proof of Proposition 6:
The mirrored operator corresponding to the optimal EW $\tilde{W}_I(\theta)$ for $\theta \in [0,\pi] - \{\pi/2\}$ can be expressed as

$$\tilde{W}_I^{\mathrm{M}}(\theta) = \frac{3}{2}\mathbb{1}_4 \otimes \mathbb{1}_4 - \tilde{W}_I(\theta) = 4\,(2-a)\,P_4^+ + B_2^\Gamma(\theta), \tag{6}$$

with

$$\begin{aligned} B_2(\theta) \;=\; & \left(\frac{3}{2}-b\right)\sum_{i=0}^{3}|i\rangle\langle i|\otimes|i+1\rangle\langle i+1| + \left(\frac{3}{2}-c\right)\sum_{i=0}^{3}|i\rangle\langle i|\otimes|i+2\rangle\langle i+2| \\ + & \left(\frac{3}{2}-d\right)\sum_{i=0}^{3}|i\rangle\langle i|\otimes|i+3\rangle\langle i+3| + \left(a-\frac{3}{2}\right)\sum_{\substack{i=0\\ i\neq\{j,j+2\}}}^{3}|i\rangle\langle j|\otimes|j\rangle\langle i| \\ + & \left(a-\frac{1}{2}\right)\sum_{\substack{i=0\\ i=j+2}}^{3}|i\rangle\langle j|\otimes|j\rangle\langle i|. \end{aligned} \tag{7}$$

Clearly, for $\theta \in [0,\pi] - \{\pi/2\}$, one has $\frac{1}{2} \leq a \leq 1,\ 0 \leq b, d \leq 1,\ 1 \leq c \leq \frac{3}{2}$. Hence, the first part of Eq. (7) is positive. Now we need to show that $B_2(\theta) \geq 0$. Note that the positivity of $B_2(\theta)$ is equivalent to the positivity of the following two 2×2 submatrices

$$A_3 = \begin{pmatrix} \frac{3}{2}-b & a-\frac{3}{2} \\ a-\frac{3}{2} & \frac{3}{2}-d \end{pmatrix} \quad \text{and} \quad A_4 = \begin{pmatrix} \frac{3}{2}-c & a-\frac{1}{2} \\ a-\frac{1}{2} & \frac{3}{2}-c \end{pmatrix}. \tag{8}$$

Simple calculation shows that $\det[A_3] = \frac{1}{2}(1-\sin\theta) \geq 0$ and $\det[A_4] = 0$ in $\theta \in [0,\pi] - \{\pi/2\}$. This proves that $B_2(\theta) \geq 0$ and hence $\tilde{W}_I^{\mathrm{M}}(\theta)$ is decomposable.
